# Early Cretaceous paleomagnetic and geochronologic results from the Tethyan Himalaya: Insights into the Neotethyan paleogeography and the India–Asia collision

**DOI:** 10.1038/srep21605

**Published:** 2016-02-17

**Authors:** Yiming Ma, Tianshui Yang, Weiwei Bian, Jingjie Jin, Shihong Zhang, Huaichun Wu, Haiyan Li

**Affiliations:** 1 State Key Laboratory of Biogeology and Environmental Geology, China University of Geosciences, Beijing 100083, China; 2 School of Earth Sciences and Resources, China University of Geosciences, Beijing 100083, China

## Abstract

To better understand the Neotethyan paleogeography, a paleomagnetic and geochronological study has been performed on the Early Cretaceous Sangxiu Formation lava flows, which were dated from ~135.1 Ma to ~124.4 Ma, in the Tethyan Himalaya. The tilt-corrected site-mean characteristic remanent magnetization (ChRM) direction for 26 sites is Ds = 296.1°, Is = −65.7°, ks = 51.7, α_95_ = 4.0°, corresponding to a paleopole at 5.9°S, 308.0°E with A_95_ = 6.1°. Positive fold and reversal tests prove that the ChRM directions are prefolding primary magnetizations. These results, together with reliable Cretaceous-Paleocene paleomagnetic data observed from the Tethyan Himalaya and the Lhasa terrane, as well as the paleolatitude evolution indicated by the apparent polar wander paths (APWPs) of India, reveal that the Tethyan Himalaya was a part of Greater India during the Early Cretaceous (135.1–124.4 Ma) when the Neotethyan Ocean was up to ~6900 km, it rifted from India sometime after ~130 Ma, and that the India-Asia collision should be a dual-collision process including the first Tethyan Himalaya-Lhasa terrane collision at ~54.9 Ma and the final India-Tethyan Himalaya collision at ~36.7 Ma.

The India-Asia collision is one of the most profound geological events of the Cenozoic, and is responsible for the uplift of the Himalayan-Tibetan plateau which has greatly influenced the climatic system^1^. A proper understanding of when, where and how did the India and Asia collide is critical for modeling the evolution of the Himalaya-Tibetan plateau and the global climate. Although many geological and geophysical investigations have been carried out in the Himalayan-Tibetan plateau in the last four decades^2^,^3^,^4^,^5^,^6^,^7^, disputes still exist concerning the amount of the India’s postulated northern extension from only a few hundred kilometers to more than 2000 km^8^, the width of the Neotethyan Ocean during the Early Cretaceous from only 1300 km^9^ to more than 6000 km^10^,^11^,^12^ or even no Mesozoic ocean along the present-day Indus-Tsangpo suture zone (ITSZ)^13^, and the India-Asia collision age ranging from 70 Ma^1^ to the Eocene/Oligocene boundary (~34 Ma)^5^, even to 25–20 Ma^14^ based on different methods.

The Himalaya terrane is subdivided into the Tethyan Himalaya, Greater Himalaya, Lesser Himalaya and Sub-Himalaya by South Tibetan detachment system (STDS), Main Central thrust (MCT), Main Boundary thrust (MBT) from north to south (Fig. 1a). Because the whole Himalaya terrane is generally regarded as the northern part of Greater India situated south of the present-day ITSZ, a traditional view on India-Asia collision is that the India craton and its postulated northern extension (Greater India) collided directly with Asia along the ITSZ^1^,^15^,^16^,^17^,^18^. Notably, Van der Voo *et al.*^10^ proposed that an intra-Neotethys subduction zone existed to the north of India during the Cretaceous by interpreting the tomographic results. Aitchison *et al.*^5^, as well as recently many other researchers^7^,^19^, suggested a dual-collision model which consists of a first India-Arc collision and a final India/Arc-Asia collision. However, some researchers^12^,^14^,^20^ recently proposed another different dual-collision model which includes a first collision of the Tethyan Himalaya with Asia at ~50–55 Ma and a final continent-continent collision of the Indian craton with the Tethyan Himalaya at ~20–25 Ma^14^ or ~40 Ma^12^,^20^.

Paleomagnetism is one of the primary methods of deciphering motion histories of terranes, and thus in principle the issues mentioned above can be solved by comparing paleogeographic positions of the Lhasa terrane, the Indian craton and the Tethyan Himalaya. For the Lhasa terrane, lots of Cretaceous paleomagnetic data from volcanics show its precollisional southern margin maintained a stable paleolatitude of ~16°N during the whole Cretaceous^12^,^17^,^21^,^22^. For the Indian craton, its paleolatitude evolution can also be well constrained by its apparent polar wander paths (APWPs)^23^,^24^. For the Tethyan Himalaya, some paleomagnetic studies^12^,^15^,^16^,^25^,^26^,^27^,^28^,^29^ have been carried out on the Cretaceous and paleocene rocks, but only a few studies yielded reliable characteristic remanent magnetization (ChRM) directions due to serious remagnetization. Moreover, three high-quality paleomagnetic results show that the northward extension of Greater India ranges from ~200 km during the Early Cretaceous^12^ to more than 1500 km during the Late Cretaceous and paleocene^15^,^16^. This difference has been explained as the occurrence of Late Cretaceous extension between the Indian craton and Tethyan Himalaya^12^,^14^,^20^, which has since been hotly debated^6^.

Noticeably, although the Cretaceous paleomagnetic data from the Tethyan Himalaya are a key to understanding the Neotethyan paleogeograpy and the India-Asia collision process, only two Cretaceous paleomagnetic data sets reported by Patzelt *et al.*^15^ and Yang *et al.*^12^ provide robust field tests. Therefore, high-quality Cretaceous paleomagnetic data are still necessary. Considering that the sedimentary rocks often suffer from compaction-induced inclination shallowing^20^,^30^,^31^,^32^, whereas the volcanic rocks are immune from its effect, we carried out a combined geochronologic and paleomagnetic study on the Early Cretaceous Sangxiu Formation (Fm) lava flows in the Tethyan Himalaya. These new high-quality and well-dated paleomagnetic data can significantly attribute to the two issues mentioned above.

The Sangxiu Fm, which is defined as an intercalated volcanic-sedimentary sequence, is only distributed in the southeast of the Yangzhuoyongcuo Lake along the eastern part of central Tethyan Himalaya (Fig. 1b). It conformably overlies the Upper Jurassic Weimei Fm and underlies the Lower Cretaceous Jiabula Fm [1:250,000 scale Luoza county regional geological survey report (H46C004001), 2002]. The age of the Sangxiu Fm volcanics is ~133 Ma indicated by SHRIMP U-Pb zircon dating^33^. The earliest folding of the Sangxiu Fm occurred in the latest Early Cretaceous [H46C004001, 2002]. The sedimentary strata include coarse to fine-grain quartz sandstone, quartz greywacke, siltstone and shale. The volcanic strata consist of pillowed and massive, sparsely amygdaloidal basalts and minor dacites.

A total of 32 paleomagnetic sites were sampled from two sections located in the southeast of the Yangzuoyongcuo Lake (Fig. 1b) and ~150 km northwest of the sampling area of the Lakang Fm lava flows^12^. Twenty-three sites were collected from section A located at (28.8°N, 91.3°E), and another 9 sites were sampled from monoclinal strata of section B at (28.8°N, 91.1°E). Each sampling site spans several meters of stratigraphic thickness and covers at least one lava flow. The bedding attitudes of both sampling sections are obvious and can be well determined by measuring the intercalated sedimentary rocks (Supplementary Fig. S1). Furthermore, two block samples of fresh volcanic rocks from sites ZL1 and ZL23 of section A were collected for zircon U-Pb chronology.

## Results

### U-Pb Zircon Geochronology

The zircon grains are euhedral to subhedral prism (50–200 μm in length) with an aspect ratio of ~1–3 (Supplementary Fig. S2). This, together with clear oscillatory zonings in Cathodoluminescence images, indicates a magmatic origin. Oscillatory zonings where without inclusions and cracks were selected and analyzed for each sample. Zircon U-Pb analyses yielded diverse age groups, indicating different sources for the zircons (Fig. 2). The weight mean ^206^Pb/^238^U ages of the main population are interpreted as the time of emplacement of the studied volcanics. Other ages of minor populations, which include less than 5 zircon grains, may yield from inherited or contaminative zircons. Samples ZL1 and ZL23 yield weighted mean ^206^Pb/^238^U ages of 135.1 ± 0.7 Ma and 124.4 ± 0.7 Ma, respectively. These new ages are well consistent with the age of ~133 ± 3 Ma reported by Zhu *et al.*^33^, indicating that the Sangxiu volcanics erupted during the Early Cretaceous. We used ~135.1–124.4 Ma for the Sangxiu Fm in further discussion.

### Rock magnetic results

Isothermal remanent magnetization (IRM) acquisition curves of the representative specimens rise very quickly below 200 mT and the saturation is essentially reached at ~160–300 mT, revealing that low-coercivity magnetic carriers are dominant (Supplementary Fig. S3a,b). Progressive demagnetization of the saturation IRM (SIRM) by applying reverse fields indicates that the maximum coercive force is less than 60 mT. This, together with a clear Curie temperature at ~580 °C, indicates that the low-coercivity magnetite is dominant in the Sangxiu Fm volcanic samples (Supplementary Fig. S3c,d). Hysteresis loops close in a magnetic field at ~250 mT, also indicating relatively low coercivity (Supplementary Fig. S4a,b). The Day plot^34^ reveals that the magnetic grains are located within the region of pseudo-single-domain (PSD) (Supplementary Fig. S4c). First-order reversal curves (FORCs)^35^ show closed peak structures with relatively open contours, suggesting the presence of PSD magnetite and minor single-domain (SD) magnetite (Supplementary Fig. S4d,e). Both PSD and SD grains are very efficient carriers of remanent magnetization, so they should most probably carry a stable remanence when the Sangxiu Fm lava flows were erupted.

### Paleomagnetic Results

About 80% of specimens underwent stepwise thermal demagnetization, and about 20% were performed stepwise alternating field (AF) demagnetization. Both demagnetization methods yield the same ChRM directions (Fig. 3). Some volcanic specimens give a low-temperature component (LTC) below ~250 °C or a low-coercivity component (LCC) below ~20 mT. Both LTC and LCC in geographic coordinates are closed to the present-day geomagnetic field direction. After removing the LTC or LCC, a high-temperature component (HTC) or a high-coercivity component (HCC) can be isolated from most specimens, which is defined as the ChRM directions, between ~400 °C and ~580 °C or between ~30 mT and ~100 mT. The ChRM directions decay toward the origin and include antipodal normal and reverse polarities (Fig. 3). However, some specimens from sites ZL6, ZL16, ZL17 and ZL18 display erratic demagnetization patterns (Fig. 3p), and no reliable ChRM directions can be isolated from them. Based on the following filtering criteria to ChRM and site-mean directions: (1) all ChRM directions are determined using principal component analysis^36^; (2) ChRM directions have maximum angular deviation values (Fisher’s precision parameter^37^) <15°; (3) site-mean directions include at least 5 samples; and (4) site mean directions have k-values > 50, twenty-eight of 32 paleomagnetic sites provide reliable site-mean directions listed in Table 1 and the corresponding ChRM directions of 230 specimens are presented in Supplementary Table S1. Notably, the tilt-corrected ChRM directions of sites ZL4 and ZL8 show >45° angular deviations from the overall-mean direction (Table 1), indicating that they probably recorded a transitional or excursional paleomagnetic direction. Therefore, these two sites are discarded for final analysis. Finally, twenty-six paleomagnetic sites yield an overall-mean direction of Dg = 268.7°, Ig = −47.4°, kg = 1.9, α_95_ = 30.0° *in situ* and Ds = 296.1°, Is = −65.7°, ks = 51.7, α_95_ = 4.0° after tilt correction (Table 1, Fig. 4). This overall-mean direction passes both McElhinny^38^ and McFadden^39^ fold tests at the 95% and 99% confident level, which, combined with that the reversal test is also positive at the 95% confidence level^40^ (Table 1), indicates that the ChRMs have a pre-folding origin and are probably primary magnetization acquired during the eruption of the Sangxiu Fm lava flows. The Fisherian site-mean paleopole for 26 sites is located at 5.9°S, 308.0°E with A_95_ = 6.1°, corresponding to a paleolatitude of 48.5° ± 6.1°S for the study area (28.8°N, 91.3°E).

Paleomagnetic data using for paleogeographic and tectonic reconstructions must average paleosecular variations. Based on the following evidences: (1) the sampling of lava flows spans a long time from ~135.1 Ma to ~124.4 Ma as determined by U-Pb zircon ages; (2) the sampling sections span many lava flows interbedded with sedimentary rocks; (3) the ChRM directions include antipodal dual polarities; (4) twenty-six lava sites provide a virtual geomagnetic pole (VGP) scatter of 17.1° at ~48.5°S, which matches with the paleosecular variation model at similar paleolatitude^41^; (5) the A_95_, which is obtained from the VGPs of 26 lava sites, is 6.1°, which is well consistent with a *N*-dependent A_95_ envelope with a 95% confidence interval (3.3°, 10.5°) proposed by Deenen *et al.*^42^, we confidently conclude that the Fisherian site-mean pole (5.9°S, 308.0°E with A_95_ = 6.1°) obtained from the 26 Sangxiu Fm lava flows has averaged paleosecular variation. Therefore, it should be a reliable Early Cretaceous pole for the Tethyan Himalaya.

## Discussion

**Figure 1:**
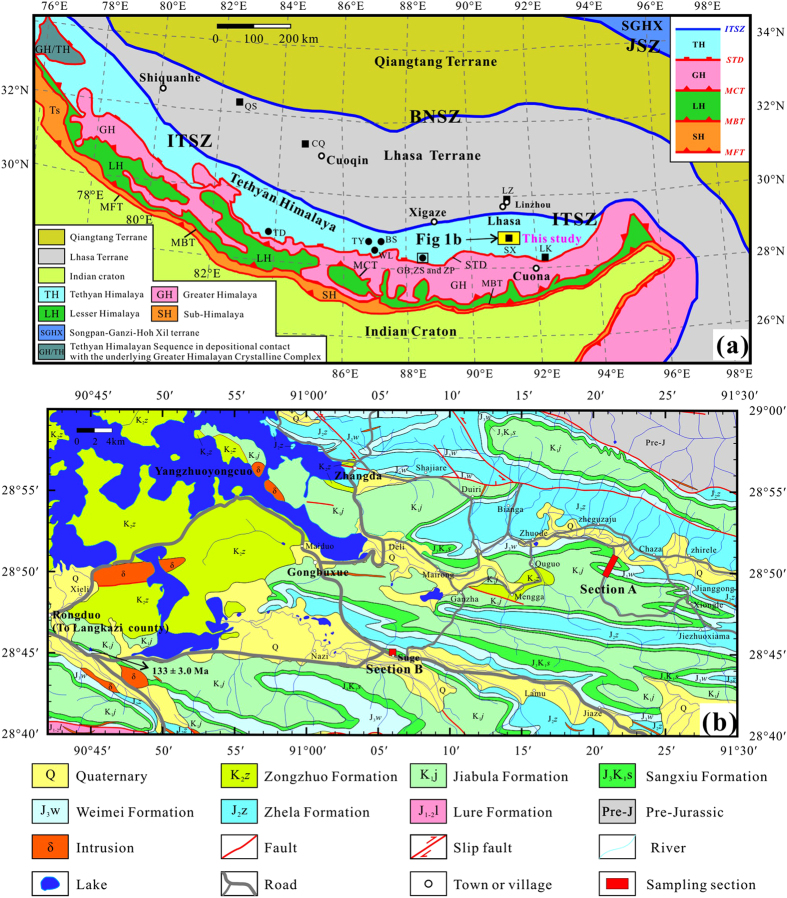
Sketches of geology and sampling location for this study. (**a**), Regional geologic map of the Himalayan belt and adjacent areas modified from Yin and Harrison^1^ and Yin^60^. Solid squares and circles show sampling locations of previous Cretaceous and Paleocene paleomagnetic studies on volcanic and sedimentary rocks, respectively (for sample location abbreviations see Table 2). Abbreviations: JSZ, Jinsha suture zone; BNSZ, Bangong–Nujiang suture zone; ITSZ, Indus–Tsangpo suture zone; STD, South Tibet detachment system; MCT, Main Central thrust; MBT, Main Boundary thrust; MFT, Main Frontal thrust. (**b**), Simplified geological map of the sampling area.

**Figure 2:**
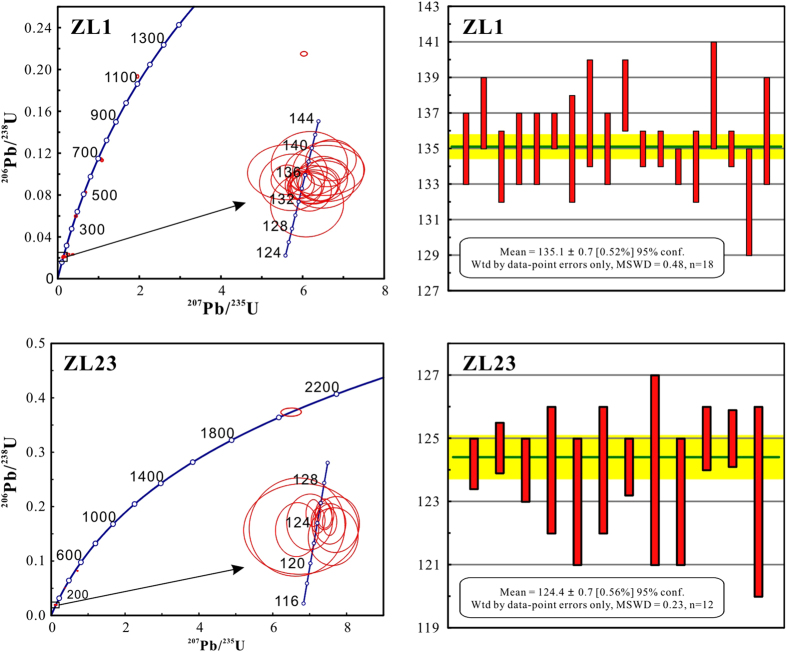
Concordia diagrams showing ^206^Pb/^238^U ratios in zircon grains in samples ZL3 and ZL8 (left columns); weighted average of apparent ^206^Pb/^238^U ages (right columns).

**Figure 3:**
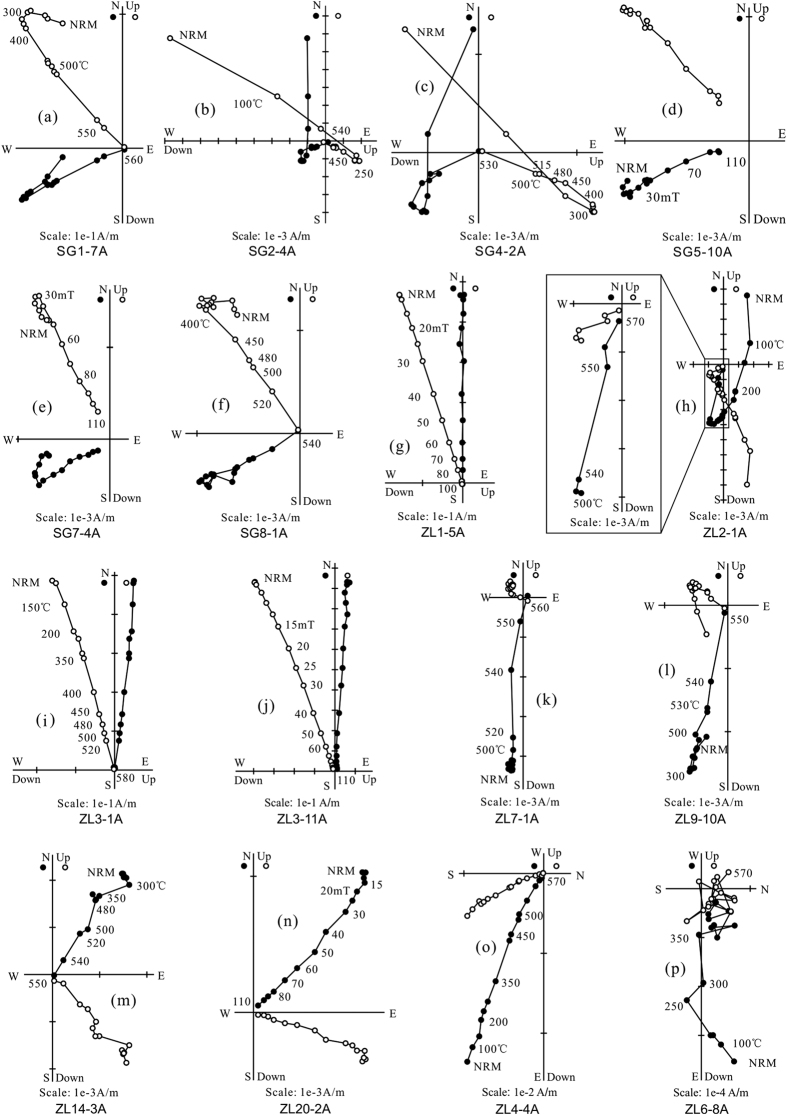
Thermal/alternating field demagnetization diagrams for representative Sangxiu Fm lava specimens in geographic coordinates. The solid and open symbols represent the projections onto the horizontal and vertical planes, respectively.

**Figure 4:**
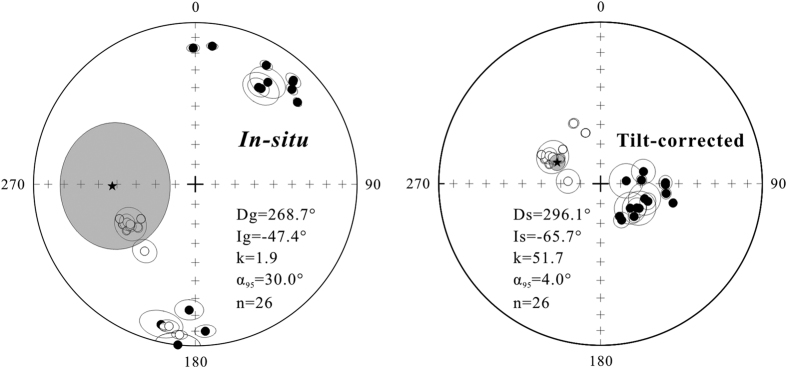
Equal-area projections of site-mean directions. The stars indicate the overall-mean direction of 26 sites.

The present geological boundary between India and Asia is along relatively east-west ITSZ that separates the Tethyan Himalaya from the Lhasa terrane (Fig. 1a), where the Neo-Tethyan Ocean opened in the Late Triassic and reached its greatest width in the Early Cretaceous^1^. Therefore, the paleolatitude evolutions of the Indian craton, the Tethyan Himalaya and the Lhasa terrane have been widely used to constrain the Cretaceous-Paleogene paleogeography of the Neotethyan Ocean and the India-Asia collision process^12^,^14^,^17^,^43^. Because the present-day ITSZ stretches a length of ~2000 km from east to west (Fig. 1a), a reference point (29°N, 87.5°E) located in its middle part has been used to calculate the expected and observed paleolatitudes (Table 2).

For the Lhasa terrane, considering that 1) the possible inclination shallowing is still a critical and unresolved problem for the validity of paleomagnetic data from sedimentary rocks^20^,^30^,^31^,^32^; 2) the Paleogene paleomagnetic data are mainly from the Linzizong Gp volcanic rocks which span a long time range from ~69 to ~40 Ma and might be erupted after the Tethyan Himalaya-Lhasa terrane collision age (such as the Panna Fm volcanic rocks)^31^,^44^,^45^; and 3) the Lhasa terrane accreted onto the Qiangtang terrane by the Early Cretaceous and its southern margin maintained a stable paleolatitude during the whole Cretaceous^11^,^12^,^17^,^20^,^43^, in this study we use two reliable Cretaceous volcanic paleomagnetic poles, which come from a large number of volcanic sites with clear bedding attitudes and satisfy all the 7-point data quality criteria proposed by Van der Voo^46^, to constrain the paleolatitude of the Lhasa terrane as Huang *et al.*^47^ recently have done. Two Early Cretaceous volcanic poles from Yanhu (QS)^17^ and Cuoqin (CQ)^11^,^20^ areas yield paleolatitudes of 18.3° ± 2.1°N and 14.9° ± 5.5°N for the reference point (29°N, 87.5°E), respectively (Table 2), and their mean paleolatitude of 16.6° ± 5.9°N should be a credible estimate for the precollisional southern margin of Asia.

For the Tethyan Himalaya, only nine Cretaceous-Paleocene poles were available due to serious remagnetization (Table 2). Three poles WL^29^, TD^25^ and GB^15^ from the Early Cretaceous sedimentary rocks don’t provide a robust field test, and two Paleocene poles BS^26^ and TY^27^ are from too small sites (specimens) to pass the basic selection criterion of specimen (site) number. Therefore, these five poles are discarded for further discussion. The remaining two volcanic (LK^12^ and SX (this study)) and two limestone (ZS^14^,^15^ and ZP^14^,^15^,^16^) poles satisfy the 7-point data quality criteria proposed by Van der Voo^46^. Noticeably, Dupont-Nivet *et al.*^48^ applied the Elongation/Inclination (E/I) correction method to these limestone paleomagnetic data from the southern Tethyan Himalaya, and suggested that they did not suffer from significant inclination shallowing. Therefore, in this study we use these four paleomagnetic poles to position the Tethyan Himalaya.

**Figure 5:**
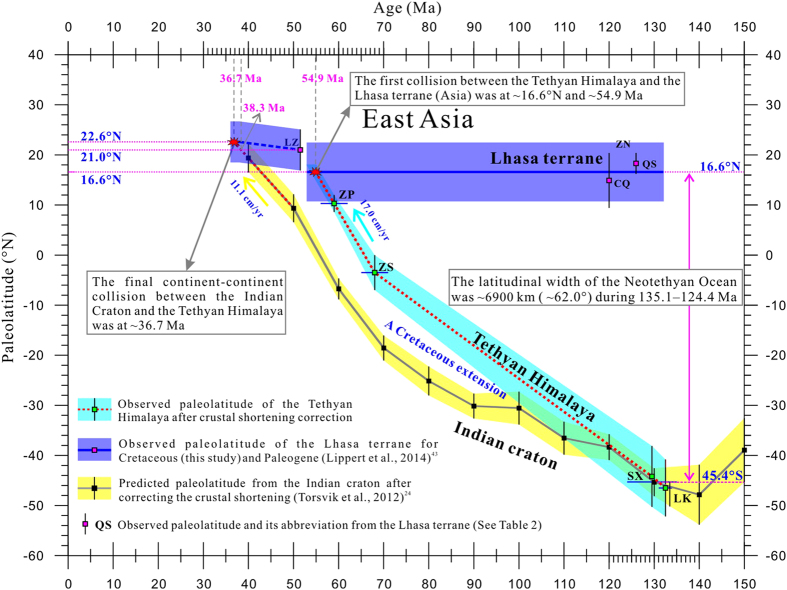
Paleolatitude evolution of the Lhasa terrane, the Tethyan Himalaya, and the Indian craton. The shaded areas and vertical bars show the errors of the paleolatitudes. Expected and observed paleolatitudes were calculated for the reference point at 29.0°N, 87.5°E.

Two Early Cretaceous volcanic poles of SX and LK yield paleolatitudes of 45.3° ± 6.1°S at ~135.1–124.4 Ma and 48.1° ± 5.7°S at ~134–131 Ma for the reference point (29°N, 87.5°E), respectively (Table 2). Considering that a minimum of 176 km (~1.6° of latitude) north-south horizontal shortening has occurred within the Tethyan Himalaya (112 km) and the ITSZ (64 km)^49^, we made a 1.6° N–S shortening correction to the LK dataset whose sampling area is located at the southern margin of the Tethyan Himalaya (Fig. 1a). Because our sampling area is located at the middle Tethyan Himalaya, a 1.1° (~120 km) N–S shortening was corrected for the SX dataset. Such the N–S shortening corrections would relocated the northern margin of the Tethyan Himalaya at a more northerly paleolatitude of 46.5° ± 5.7°S at ~134–131 Ma and 44.2° ± 6.1°S at ~135.1–124.4 Ma (Fig. 5). Considering that these two paleolatitudes are very consistent within the paleomagnetic confidence level, as well as that the age range of the SX pole entirely covers that of the LK pole, we use their mean paleolatitude of ~45.4°S as a credible estimate for the Tethyan Himalaya at the reference point (29°N, 87.5°E) (Fig. 5). Comparing it with ~16.6°N for the southern margin of the Lhasa terrane shows a paleolatitude difference of ~62.0°, implying that the Neotethyan Ocean between the Tethyan Himalaya and the Lhasa terrane opened a latitudinal width up to ~6900 km during ~135.1–124.4 Ma (Fig. 5). This width is also consistent with ~6700 km and ~7000 km estimated by Chen *et al.*^11^ and Yang *et al.*^12^, respectively.

Comparing the Early Cretaceous (135.1–124.4 Ma) paleolatitude of ~45.4°S observed from the Tethyan Himalaya with the ~49.6°S and ~50.2°S predicted by the most widely used^23^ and most recent global synthetic^24^ Indian APWPs at 130 Ma for the reference point (29.0°N, 87.5°E) indicates a paleolatitude difference of ~4.2° (~470 km) and ~4.8° (~530 km), respectively. Although the ~4.2°–4.8° (~470–530 km) crustal shortening deduced from high-quality Early Cretaceous paleomagnetic data is within the paleomagnetic resolution (~6° or ~670 km), the most possible crustal shortening amount (~470–530 km) deduced from these high quality paleomagnetic results is basically consistent with ~480–650 km, which includes ~176 km within the Tethyan Himalaya and the ITSZ^49^ and ~302–476 km between the MFT and the STDS^50^, estimated by balanced cross-section analyses, supporting that the Tethyan Himalaya belonged to a contiguous Indian continental lithosphere at 130 Ma^12^,^14^,^29^.

Based on the Late Cretaceous and Paleocene paleomagnetic data^15^,^16^ reanalyzed by van Hinsbergen *et al.*^14^, the Tethyan Himalaya was located at paleolatitudes of 5.1° ± 3.5°S at ~68 Ma and 8.7° ± 1.7°N at ~59 Ma for the reference point (29°N, 87.5°E), respectively (Table 2). A ~176 km (~1.6° of latitude) N–S shortening correction^49^ would relocated the northern margin of the Tethyan Himalaya at paleolatitudes of 3.5° ± 3.5°S at ~68 Ma and 10.3° ± 1.7°N at ~59 Ma (Fig. 5). Comparing them with the two coeval paleolatitudes of ~21.0°S and ~10.0°S calculated from the Indian APWPs^24^ reveals a paleolatitude difference of ~17.5° (~1940 km) for ~68 Ma and ~20.3° (~2250 km) for ~59 Ma, respectively. Obviously, the paleolatitude difference observed from the high-quality Late Cretaceous and Paleocene data is much greater than ~4.8° (~530 km) determined by the high-quality Early Cretaceous (~135.1–124.4 Ma) volcanic data and ~480–650 km estimated by balanced cross-section analyses^49^,^50^, as well as far more than the Indian subcontinent extension of ~500–950 km deduced from the fitting of India in-Gondwana and an analysis of bathymetric features in the eastern Indian Ocean^8^. Therefore, such a great paleolatitude gap between the Indian craton and the Tethyan Himalaya cannot wholly be attributed to the continental crustal shortening within the Himalaya terrane after the India-Asia collision^15^,^16^,^17^,^51^, as well as to an ocean existed between the Indian craton and the Tethyan Himalaya even earlier in the Early Cretaceous^13^ and it subducted beneath the Tethyan Himalaya after the Eocene. However, it can be interpreted as a small post-Neotethyan ocean (basin) extended between the Indian craton and the Tethyan Himalaya after the Early Cretaceous, and it subducted beneath the Tethyan Himalaya after the Eocene^12^,^14^,^20^. Such a Cretaceous extension model implies that the India-Asia collision should be a dual-collision process, which consists of a first collision occurred between the Tethyan Himalaya and the Lhasa terrane and a final collision occurred between the India craton and Tethyan Himalaya (Fig. 5).

The collisional age of the Tethyan Himalaya and the Lhasa terrane can be determined by their paleolatitudes overlaps. Based on high-quality Late Cretaceous and Paleocene paleomagnetic data observed from the Tethyan Himalaya, it moved northward at a velocity of ~17.0 cm/yr during ~68–59 Ma. Extrapolating the constant northward velocity ~17.0 cm/yr implies that the Tethyan Himalaya would intersect with the Lhasa terrane at ~54.9 Ma (Fig. 5). Lippert *et al.*^43^ compiled reliable paleomagnetic results from the upper Linzizong Fm (51.5 ± 4.5 Ma) volcanics. Based on applying the strict filtering criteria to site-mean directions, they obtained a paleomagnetic pole (80.2°N, 230.4°E with A_95_ = 4.1°) from 41 lava sites, This pole yields a paleolatitude of 21.0° ± 4.1°N for the reference point (29.0°N, 87.5°E), which should be a reliable estimate for the southern margin of the Tethyan Himalaya-Lhasa terrane at 51.5 ± 4.5 Ma. Assuming that the crustal shortening within the Himalaya terrane occurred after the second collision, a 4.8° (~530 km) N–S crustal shortening correction would relocate the reference point of the India Craton at paleolatitudes of ~9.3°N at 50 Ma and ~19.3°N at 40 Ma, implying that the India Craton moved northward at an average velocity of ~11.1 cm/yr between 50 Ma and 40 Ma. Extrapolating the velocity of ~11 cm/yr suggests that the India Craton would collide with the Tethyan Himalaya at ~38.3 Ma.

Because the crustal shortening in Asia is ~600–750 km in the last 50 Ma^52^ (i.e. ~12–15 km/Myr), the shortening amount for the time interval of ~50–38 Ma can be up to ~144–180 km (~1.3–1.6° of latitude). We conservatively add 1.6° of latitude to the leading edge of the Tethyan Himalaya-Lhasa terrane for ~38–36 Ma to estimate the effect of postcollisional shortening within Asia (Fig. 5). A final collision between the India Craton and the Tethyan Himalaya-Lhasa terrane was occurred at ~22.6°N at ~36.7 Ma (Fig. 5). It is important to note that dating the second collision largely depends on the paleolatitude observed from the upper Linzizong Fm volcanics and crustal shortening in the Asia and the Himalaya terrane prior to the second collision.

Critically, such a two-stage collision model also match with many geological evidences, such as: (1) the northward drift velocity of the Indian craton sharply decreases at ~55 Ma^3^,^4^ and ~40 Ma^24^; (2) a small post-Neotethyan Ocean opened during the Late Cretaceous and then subducted at the time interval of ~55–37 Ma, which corresponds to a two-stage collision model^12^,^20^, can explain the reason why the ‘missing’ convergence didn’t be documented by geological record of Asia and the Himalaya^14^; (3) the Asian assemblage arrived on the Tethyan Himalaya during the Early Eocene^53^, while the Tethyan Himalaya detritus reached the Indian foreland region during the Middle Eocene^6^; (4) the subduction-related ultrahigh-pressure rocks in the Himalayan terrane dated at the Early Eocene^54^, while the earliest deep tectonic burial, high-grade metamorphism, and anatexis in the Greater Himalaya and middle-southern Tethyan Himalaya occurred during the middle Eocene^55^,^56^.

In summary, we have obtained a high quality and well-dated Early Cretaceous (135.1–124.4 Ma) paleopole (5.9°S, 308.0°E with A_95_ = 6.1°) for the Tethyan Himalaya. This new paleomagnetic pole not only satisfies the secular variation model, but fulfills all the seven quality criteria proposed by Van der Voo^46^ to appraise the reliability of paleomagnetic data. Based on our new paleomagnetic data and previous high-quality Cretaceous and Paleocene paleomagnetic results from the Tethyan Himalaya and the Lhasa terrane, as well as the APWPs of the India craton, several main conclusions can be summarized as follows: (1) the Gongbuxue area of the Tethyan Himalaya during ~135–124 Ma was located at ~48.5°S; (2) the Tethyan Himalaya belonged to a contiguous Indian continental lithosphere at ~130 Ma and it ever separated from the Indian Craton sometime after ~130 Ma; (3) in the present-day Indian craton coordinates the Neotethyan Ocean between the Indian craton and the Tethyan Himalaya was up to a latitudinal width of ~6900 km (~62.0°) during ~135–124 Ma; (4) the India-Asia collision should be a dual-collision process including the first Tethyan Himalaya-Lhasa terrane collision occurred at ~54.9 Ma and the final India-Tethyan Himalaya collision occurred at ~36.7 Ma.

## Methods

Six paleomagnetic sites were collected with standard paleomagnetic cores from section A using a portable gasoline-powered drill and were oriented by both a magnetic compass and a Sun compass. A declination difference is less than 2° by comparing these two oriented results, indicating that the local magnetic disturbance can be neglected. Twenty-six paleomagnetic sites were collected with oriented blocks because the portable drills were broken during the field sampling. Cores (25 mm diameter) were further drilled from the block samples in the laboratory.

Standard 2.5-cm-diameter cores were cut into 2.2-cm-long specimens in the laboratory. Most specimens were subjected to either stepwise thermal demagnetization from 100 °C to ~580 °C in an ASC-TD 48 furnace with an internal residual field less than 10 nT or alternating field (AF) demagnetization from 5 mT to 110 mT using a D-2000 alternating field demagnetizer. Remanent magnetization measurements were carried out with 2G-755-4K cryogenic magnetometers. All stepwise demagnetization and remanent magnetization measurements were performed in a shielded room with residual fields less than 300 nT at the Paleomagnetic and Environmental Magnetism Laboratory (PEML) at the China University of Geosciences, Beijing (CUGB). ChRM directions of all the specimens were determined using principal component analysis^36^, and site-mean directions were calculated using Fisherian statistics^37^. Paleomagnetic data were analyzed using Enkin’s^57^ and Cogné’s^58^ computer program packages.

Acquisition of the IRM, backfield demagnetization of SIRM, thermal demagnetization of the three-axis IRM, hysteresis loops, and FORCs were performed on some representative standard specimens and corresponding powder specimens. The IRM, backfield demagnetization of SIRM and three-axis IRM were acquired using an IM10-30, and then were measured using a JR-6A spinner magnetometer at PEML of the CUGB. Hysteresis loops, FORCs and magnetic hysteresis parameters including saturation magnetization (Ms), saturation remanent magnetization (Mrs), coercivity (Bc), remanence coercivity (Bcr) were measured on representative powder samples at room temperature using a MicroMag Model 3900 Vibrating Sample Magnetometer at the Institute of Geophysics, China Earthquake Administration. FORCs’ data were processed using the FORCinel software with a smoothing factor (SF) of 6^35^.

Zircons and images preparation are following the methods described in Ma *et al.*^17^. U-Pb zircon geochronology was performed using a laser-ablation-multicollector inductively coupled-plasma-mass (LA-ICP-MS) at the Institute of Tibetan Plateau Research, Chinese Academy of Sciences. More detailed analytical procedures and configuration of the LA-ICP-MS have been described in Wu *et al.*^59^.

## Additional Information

**How to cite this article**: Ma, Y. *et al.* Early Cretaceous paleomagnetic and geochronologic results from the Tethyan Himalaya: Insights into the Neotethyan paleogeography and the India–Asia collision. *Sci. Rep.* **6**, 21605; doi: 10.1038/srep21605 (2016).

## Supplementary Material

Supplementary Information

## Figures and Tables

**Table 1:** Site-mean ChRM directions of the Sangxiu Fm lava flows from the Gongbuxue area in the Tethyan Himalaya.

Site ID	n/N	Strike/Dip(°)	Dg(°)	Ig(°)	Ds(°)	Is(°)	k	α_95_(°)	Devi(°)	Plat(° N)	Plon(° E)
ZL1	8/8	295/85	359.1	16.8	336	−56.4	471	2.6	19.7	20.5	291.7
ZL2	7/10	295/85	193.7	11.6	83.7	77	50.1	8.6	17.4	−28.5	299.7
ZL3	9/11	295/85	7	15	343.6	−63.2	514.4	2.3	19.8	15	283.4
*ZL4	8/8	295/85	121.6	11.7	103.8	7.5	85.9	6	57	10.2	351.3
ZL5	6/11	295/85	186.2	0	129.5	70.6	70.5	8	8.2	−4.2	297.9
ZL6	0/6	295/85	No a reliable site-mean direction								
ZL7	8/8	299/91	186	−7	134.4	66.3	353	3	7.2	2.4	299.6
*ZL8	6/7	299/91	347.2	45.6	355.5	−32.3	120.5	6.1	46.8	43.5	277.3
ZL9	10/10	299/91	192.5	−10.6	149.9	71	749.3	1.8	14	1.9	287.9
ZL10	8/8	299/91	190.5	−11.6	149.9	68.8	193.7	4	13.5	4.8	289.4
ZL11	8/10	292/80	182.8	23.1	74.2	67.5	82.2	6.1	18.4	−31.5	317.5
ZL12	8/8	292/80	176.1	9.7	110.4	64.5	184.7	4.1	3.7	−7.9	312.2
ZL13	7/8	100/68	35.4	23.6	108.8	66.5	54.6	8.2	5.1	−10.3	310.4
ZL14	7/8	100/68	34.5	28.9	122.5	66.9	51.9	8.5	3.8	−3.5	304.6
ZL15	7/10	100/68	33.1	29.2	123.8	68	144.3	5	5.0	−3.9	302.9
ZL16	0/8	100/68	No a reliable site-mean direction								
ZL17	0/9	100/68	No a reliable site-mean direction								
ZL18	0/9	100/68	No a reliable site-mean direction								
ZL19	9/9	100/68	51.2	20.1	104.9	51.7	593.9	2.1	13.8	−3.9	326.2
ZL20	10/10	100/68	43.6	13.6	90.9	57	803	1.7	15.0	−16.4	327
ZL21	8/8	100/68	30.8	15.4	85.1	69.3	429.2	2.7	14.4	−25.4	313
ZL22	8/8	100/68	43.3	12.6	89.1	57	247.6	3.5	15.8	−17.7	327.6
ZL23	9/9	100/68	45.5	17.3	98.5	56.3	364.5	2.7	12.3	−10.7	324.9
SG1	9/9	103/38	241.1	−47.6	294.2	−59.2	438.6	2.5	4.8	−2	315.5
SG2	8/9	103/38	217.1	−46.9	274.3	−73.6	85.5	6	13.3	−22.4	304.3
SG3	9/9	103/38	235.9	−47.4	290.6	-62.3	894.5	1.7	3.8	−6.3	314.1
SG4	8/8	103/38	233.2	−53.3	302.1	−65.9	438.9	2.6	2.8	−2.8	305.5
SG5	11/11	103/38	245.4	−47.3	296.2	−56.5	484.3	2.1	7.1	1.1	316.8
SG6	10/10	103/38	237.2	−50.6	297.9	−62.8	280.7	2.9	0.8	−2.4	310.4
SG7	10/10	103/38	237.5	−57.3	312.4	−64.2	483.9	2.2	6.1	3.7	302.1
SG8	8/8	103/38	240.8	−50.1	298.6	−60.4	188.2	4	3.1	−0.2	312.3
SG9	6/8	103/38	238.4	−51	299.1	−62.2	68.2	8.2	1.3	−1.3	310.5
**Overall-mean N = 26 sites**		**216/283**	**268.7**	−**47.4**	**296.1**	−**65.7**	**51.7**	**4.0**		**− 5.9**	**308.0 **

Notes: Site ID, site identification; n/N, number of samples used to calculate mean and measured; Dg, Ig, Ds, Is, declination and inclination in geographic and stratigraphic coordinates, respectively; k, the best estimate of the precision parameter; α_95_, 95% confidence limit of Fisher statistics after tilt correction; Devi means the angular deviation from the overall mean direction of 28 sites; Plat and Plon, latitude and longitude of paleopoles in stratigraphic coordinates. *Sites were not used to calculate the final mean direction.

Overall-mean (N = 26 sites without *sites):① The McElhinny fold test^38^ is positive at 95% and 99% confidence levels: ks/kg = 27.54 > F(2*(n2-1), (n1-1)) at 5% and 1% point = 1.60 and 1.94, respectively; ② The McFadden fold test^39^ is positive at 95% and 99% confidence levels. “Xi” test: critical Xi at 95% = 5.93 and at 99% = 8.39, respectively. “Xi1” and “Xi2” IS = 19.47 and 25.53, “Xi1” and “Xi2” TC = 2.83 and 0.81, respectively; ④ The reversals test^40^ is positive at 95% confidence level. Normal polarity: N1 = 11, D1 = 305°, I1 = −63.6°, k1 = 65.5; Reverse polarity: N2 = 15, D2 = 108.8°, I2 = 66.8°, k2 = 50.4; The angle between the two mean directions is γ = 7.5° < γ_critical_ = 7.8°; classification B.

**Table 2:** Summary of Cretaceous and Paleocene paleomagnetic results from the Tethyan Himalaya and the Lhasa terrane.

ID	Rock units, lithology	Area	Slat	Slon	Age	Plat	Plon	A_95_(dp/dm)	Paleolat	n/N	Test	References
(°N)	(°E)	(Ma)	(°N)	(°E )	(°)	(°N)						
Tethyan Himalaya												
*TD*	*Thakkhola-Dzong Fm, sed*	*Dzong*	*28.8*	*83.8*	*~121–112*	*12.0*	*289.0*	*6.0/7.5*	*−44.0* ± *6.0*	*95/–*	*no*	25
*WL*	*Wölong Fm, sandstone*	*Wölong*	*28.5*	*87.0*	*~120–149*	*4.4*	*256.0*	*3*	−*54.8* ± *3.0*	*201/–*	*no*	29
LK	Lakang Fm, lava	Cuona	28.1	92.4	~131–134	-26.8	315.2	5.7	−48.1 ± 5.7	225/31	F, R	12
**SX**	**Sangxiu Fm**, **volc**	**Langkazi**	**28.8**	**91.3**	**~124–135**	−**5.9**	**308.0**	**6.1**	−**45.3 ± 6.1**	**216/26**	**F**, **R**	**This study**
GB	Gamba Gp, sed	Gamba	28.3	88.5	~98–107	38.4	277.9	5.7/9.5	−21.9 ± 5.7	23*/–*	no	15
ZS	Zongshan Fm, limestone	Duela, Gamba	28.0, 28.3	89.2, 88.5	65–71	55.8	261.6	3.5	−5.1 ± 3.5	144*/–*	F, D	14,15
ZP	Zongpu, limestone	Gamba, Duela	28.3, 28.0	88.5, 89.2	56–62	69.6	272.5	1.7	8.7 ± 1.7	243*/–*	F, D	14–16
*TY*	*Zongpu, limestone*	*Dingri*	*28.7*	*86.8*	*Paleocene*	*42.6*	*280.1*	*4.0/7.3*	−*17.5* ± *4.0*	*15/3*	F*	27
*BS*	*Zongpu, limestone*	*Dingri*	*28.7*	*87.2*	*Paleocene*	*50.6*	*307.9*	*5.3/10.6*	−*2.8* ± *5.3*	*28/4*	*F*	26
Lhasa terrane												
CQ	Zenong Gp/Dianzhong Fm, volc	Cuoqin	31.3	84.8	~120	64.9	328	5.5	14.9 ± 5.5	278/30	F, D	11,20
QS	Qushenla Fm, lava	Yanhu	32.3	82.6	~120–132	61.4	192.9	2.1	18.3 ± 2.1	444/51	F, D	17
LZ	Linzizong Gp volc	Penbo	30.0	91.1	~51.5	80.2	230.4	4.1	21.0 ± 4.1	-/40	F, D	43

Notes: ID, paleopoles abbreviation used in the plot and text; Fm, Formation; Gp, Group; volc, volcanics; Slat and Slon, latitude and longitude of the sampling area; Plat and Plon, latitude and longitude of the pole; A_95_, the radius that the mean pole lies within 95% confidence; dp/dm, semi-axes of elliptical error of the pole at a probability of 95%; Paleolat, paleolatitude calculated for the reference point at 29°N, 87.5°E; n/N, number of samples or sites used to calculate Fisher mean; F means a positive fold test; R means a positive reversal test and D means dual-polarity ChRM direction; F* means a positive fold test with additional data from the adjacent sampling area.
