# Supplementary material for: Early Cretaceous paleomagnetic and geochronologic results from the Tethyan Himalaya: Insights into the Neotethyan paleogeography and the India–Asia collision
Source: Sci Rep. 2016 Feb 17;6:21605. doi: 10.1038/srep21605 (PMC4756285; doi:10.1038/srep21605)
Supplement: Supplementary Information [file srep21605-s1.pdf]

# Supplementary Information

## Early Cretaceous paleomagnetic and geochronologic results from the Tethyan Himalaya: Insights into the Neotethyan paleogeography and the India–Asia collision

Yiming Ma[a,b], Tianshui Yang[a,b,]*, Weiwei Bian[b], Jingjie Jin[b],
Shihong Zhang[a,b], Huaichun Wu[a], Haiyan Li[a]

**Supplementary Figure S1**

**Supplementary Figure S2**

**Supplementary Figure S3**

**Supplementary Figure S4**

**Supplementary Table S1**

**Supplementary Figure S1**

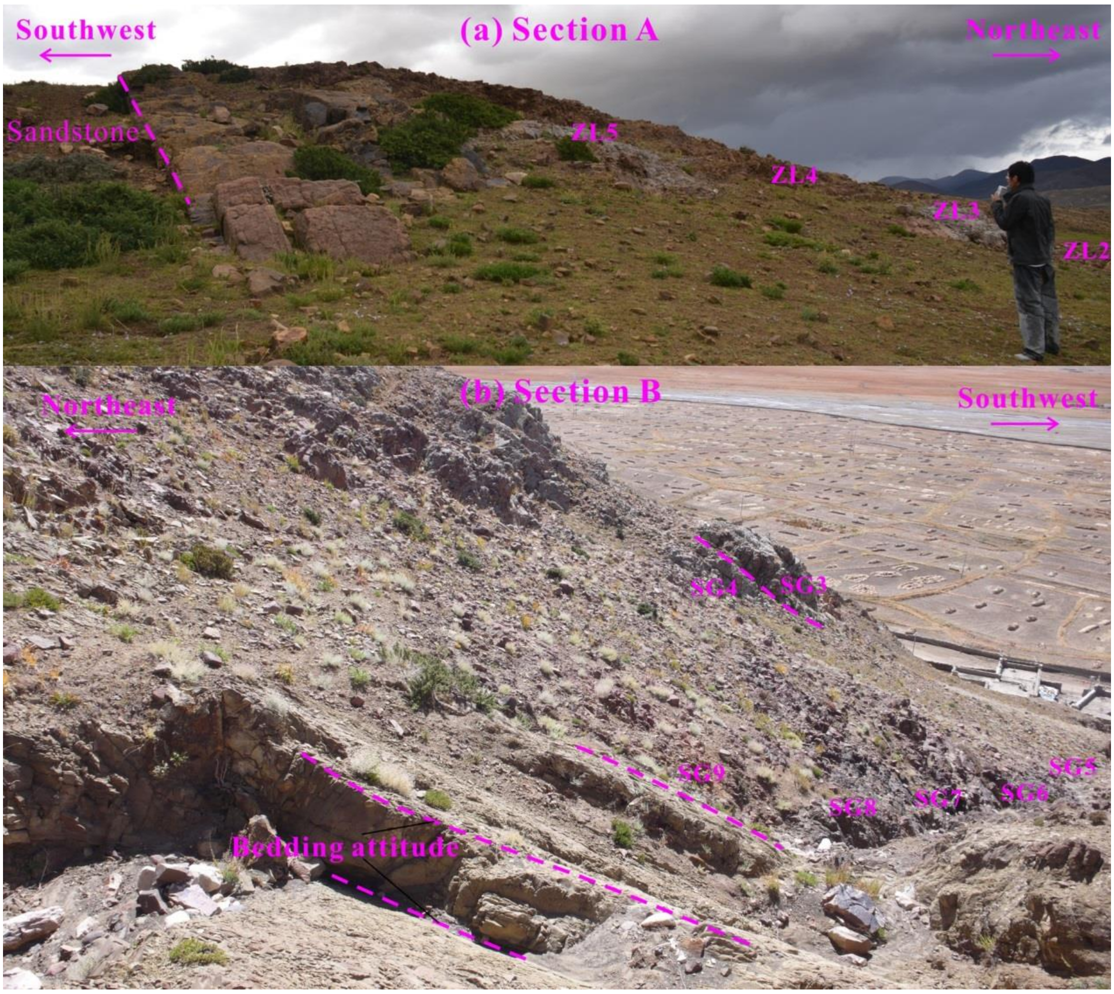


**Supplementary Figure S1.** Photographs showing the field outcrops in the studied section.

**Supplementary Figure S2**

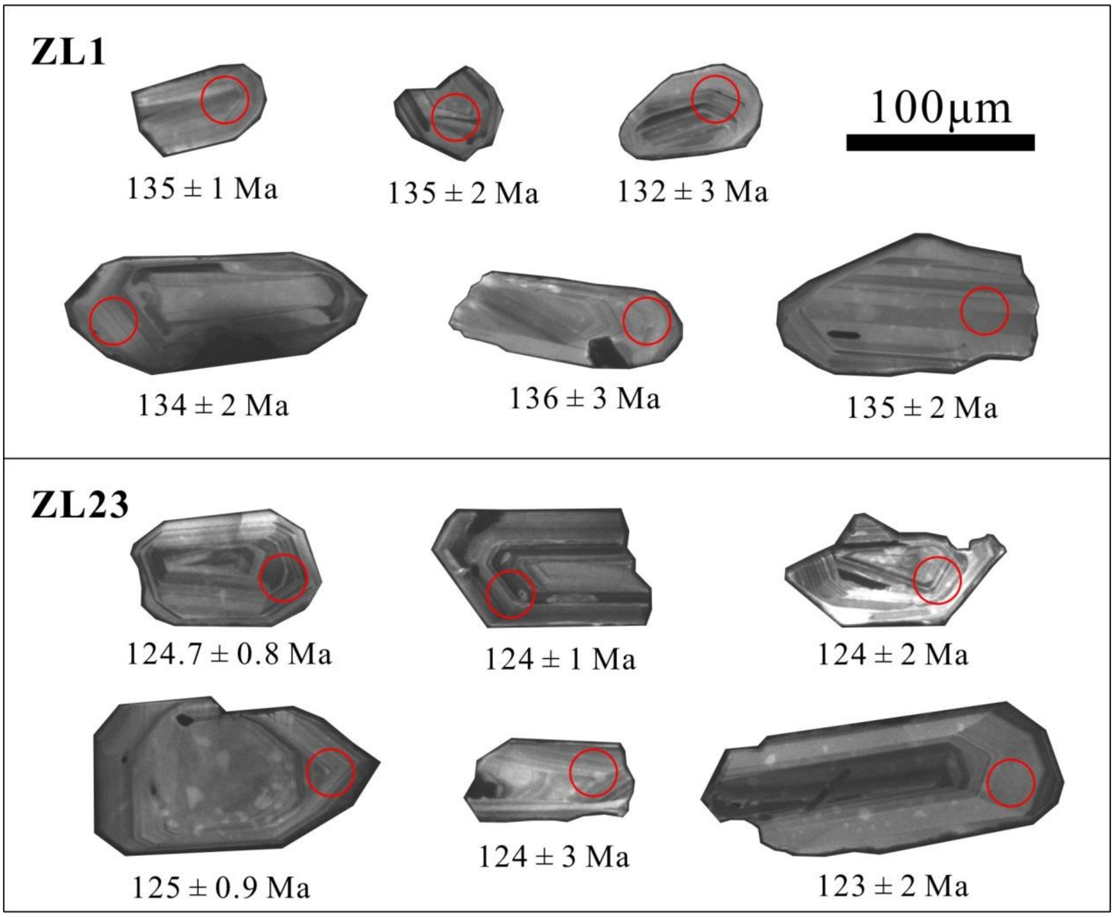


**Supplementary Figure S2.** Cathodoluminescence images of representative zircons of samples ZL3 and ZL8 and corresponding $^{206}Pb/^{238}U$ ages of the individual analyzed spots.

**Supplementary Figure S3**

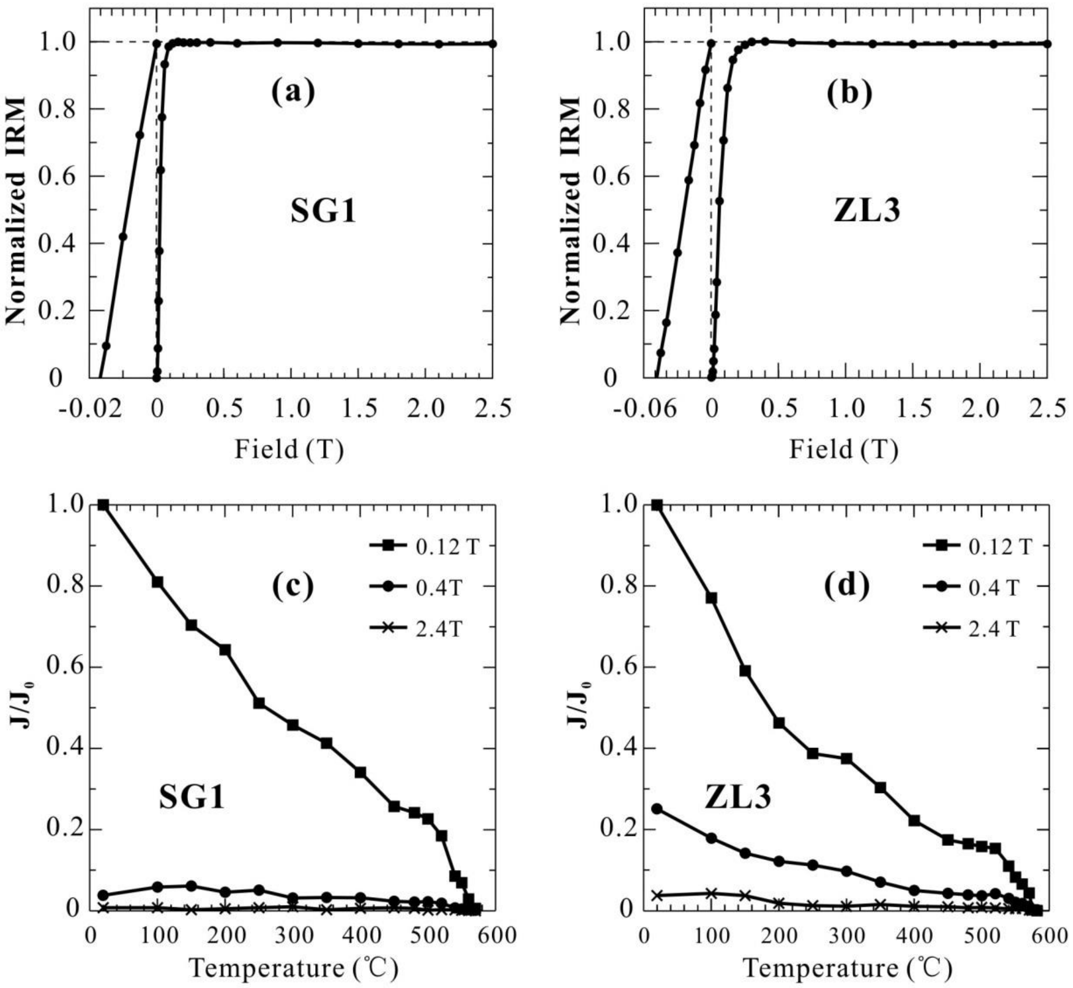


**Supplementary Figure S3.** Normalized isothermal remanent magnetization (IRM) acquisition curves and opposite field demagnetization (a, b) and thermal demagnetization of three-component IRM (c, d) for the representative specimens.

**Supplementary Figure S4**

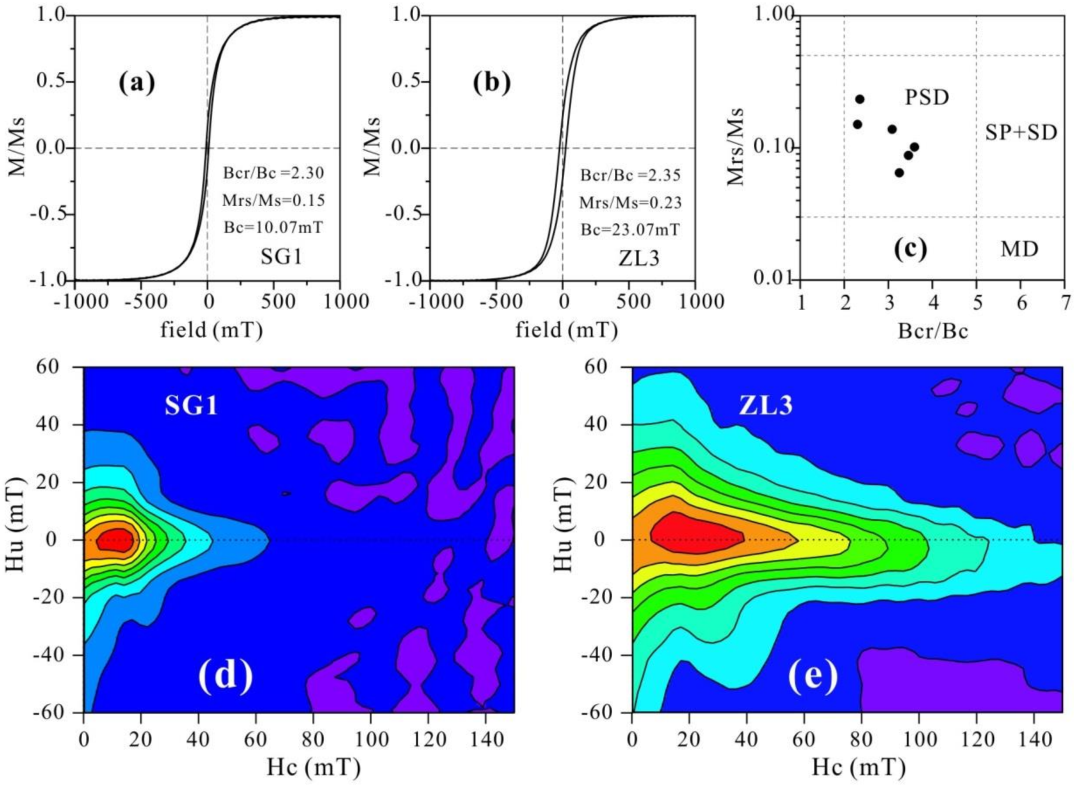


**Supplementary Figure S4.** Hysteresis loops (a, b), Day plot (c), and FORC diagrams (d, e) for the representative samples.

**Table S1.** Characteristic remanent magnetization (ChRM) directions from 28 volcanic sites of the Sangxiu Fm.

| ID | Dg | Ig | Ds | Is | MAD |
|---|---|---|---|---|---|
| SG1-1A | 240 | -50.5 | 299 | -61.1 | 3.3 |
| SG1-2A | 241.1 | -48 | 295 | -59.4 | 0.4 |
| SG1-3A | 234.8 | -47 | 289.3 | -62.8 | 10.3 |
| SG1-4A | 243.4 | -47.5 | 295.4 | -57.8 | 4 |
| SG1-5A | 247.1 | -46.9 | 296.6 | -55.3 | 2.1 |
| SG1-6A | 240.7 | -44 | 287.9 | -57.7 | 0.5 |
| SG1-7A | 243.9 | -45.2 | 292 | -56.4 | 1.8 |
| SG1-8A | 232.9 | -51.4 | 297.6 | -65.6 | 12.6 |
| SG1-9A | 244.9 | -47.1 | 295.6 | -56.7 | 5.3 |
| SG2-1A | 203.4 | -48.4 | 258.7 | -82.4 | 10.1 |
| SG2-1B | 200.9 | -50 | 264.3 | -84.7 | 10 |
| SG2-2A | 229 | -41.2 | 273.5 | -63.4 | 4.7 |
| SG2-3A | 215.8 | -44.8 | 266.4 | -73.4 | 7.3 |
| SG2-3B | 209.8 | -48.4 | 271.2 | -78.7 | 4.7 |
| SG2-4A | 234.5 | -42.5 | 280.7 | -60.7 | 8.9 |
| SG2-5A | 217.2 | -49.6 | 283.6 | -74.6 | 4.5 |
| SG2-7A | 221.7 | -45.7 | 275.8 | -70.2 | 3.1 |
| SG3-1A | 234.1 | -48.8 | 292.4 | -63.9 | 10.2 |
| SG3-2A | 232.9 | -47 | 287.9 | -64 | 9.8 |
| SG3-3A | 239.4 | -45.8 | 290 | -59.5 | 3.7 |
| SG3-3B | 237.1 | -46.2 | 289.1 | -61 | 3.5 |
| SG3-4A | 238.5 | -46.8 | 291.2 | -60.4 | 6.4 |
| SG3-5A | 228.2 | -49.3 | 290.1 | -67.8 | 7.5 |
| SG3-6A | 239.5 | -47.1 | 292.4 | -60 | 2.7 |
| SG3-7A | 235.8 | -47 | 289.8 | -62.2 | 4.2 |
| SG3-8A | 237.1 | -47.7 | 292.1 | -61.7 | 11.2 |
| SG4-1A | 236.8 | -54.3 | 305.6 | -64.1 | 5.2 |
| SG4-1B | 236 | -51 | 298.2 | -63.6 | 5.8 |
| SG4-2A | 225.5 | -53.4 | 299.9 | -70.4 | 9.5 |
| SG4-3A | 230.6 | -55.6 | 307.3 | -67.8 | 9.7 |
| SG4-4A | 237.4 | -54 | 305.1 | -63.6 | 5.1 |
| SG4-5A | 225 | -55.1 | 305.1 | -70.9 | 6.2 |
| SG4-6A | 237.6 | -53.1 | 303.3 | -63.3 | 4.2 |
| SG4-7A | 236.1 | -48.9 | 293.8 | -62.8 | 6.8 |
| SG5-1A | 244.8 | -47.5 | 296.3 | -57 | 3.5 |
| SG5-1B | 246.7 | -52 | 304.8 | -57.7 | 10.1 |
| SG5-2A | 246.3 | -49.6 | 300.4 | -57 | 4.3 |
| SG5-4A | 249.5 | -46.6 | 297.5 | -53.7 | 9.5 |
| SG5-4B | 250.4 | -48.4 | 300.7 | -54 | 6.4 |
| SG5-5A | 242.4 | -45.5 | 291.6 | -57.5 | 9.3 |

| | | | | | |
|---|---|---|---|---|---|
| SG5-6A | 248 | -49.2 | 300.7 | -55.8 | 9.5 |
| SG5-7A | 247.5 | -49.8 | 301.4 | -56.4 | 8.8 |
| SG5-8A | 241.5 | -43.8 | 288.1 | -57.1 | 11.3 |
| SG5-9A | 239.7 | -43.8 | 286.9 | -58.2 | 14.2 |
| SG5-10A | 243.4 | -43.7 | 289.3 | -55.9 | 4.1 |
| SG6-1A | 240.5 | -54.3 | 306.8 | -62 | 1.6 |
| SG6-2A | 231.2 | -47.7 | 288.4 | -65.3 | 11.3 |
| SG6-3A | 247.6 | -44.7 | 293.6 | -53.9 | 2.9 |
| SG6-4A | 237.7 | -50.5 | 297.8 | -62.4 | 4.5 |
| SG6-5A | 241.2 | -52.5 | 303.4 | -61 | 8.6 |
| SG6-6A | 241.9 | -47.6 | 294.7 | -58.7 | 7.8 |
| SG6-7A | 228.3 | -51.2 | 294.9 | -68.3 | 8.7 |
| SG6-7B | 233.3 | -53.7 | 303.2 | -65.9 | 4.7 |
| SG6-8A | 235.5 | -52.6 | 301.4 | -64.4 | 6.1 |
| SG6-9A | 233.9 | -50.2 | 295.3 | -64.6 | 4 |
| SG7-1A | 224.5 | -55.1 | 305.1 | -71.2 | 12 |
| SG7-2A | 235.3 | -59.4 | 317.2 | -65.5 | 3.1 |
| SG7-3A | 234.4 | -57.4 | 312.3 | -65.9 | 1.8 |
| SG7-4A | 237.7 | -59.3 | 317.1 | -64.3 | 1.5 |
| SG7-6A | 238.6 | -57 | 312.1 | -63.6 | 7.6 |
| SG7-7A | 236 | -58 | 314 | -65.1 | 1.5 |
| SG7-8A | 244.6 | -58.3 | 316 | -60.6 | 4.2 |
| SG7-8B | 243.4 | -57.6 | 314.2 | -61.1 | 4.6 |
| SG7-9A | 242.3 | -53.3 | 305.3 | -60.7 | 6.5 |
| SG7-10A | 238.5 | -55.9 | 309.6 | -63.4 | 5.8 |
| SG8-1A | 243.7 | -48.6 | 297.5 | -58.1 | 3.2 |
| SG8-2A | 245.7 | -60.1 | 319.7 | -60.3 | 7.5 |
| SG8-3A | 227.8 | -50.6 | 293.2 | -68.4 | 5.9 |
| SG8-4A | 235.8 | -45.5 | 287.1 | -61.5 | 7.3 |
| SG8-5A | 245.9 | -49.3 | 299.8 | -57.1 | 5.6 |
| SG8-6A | 243.5 | -47.3 | 295.2 | -57.6 | 5 |
| SG8-7A | 240.9 | -48.2 | 295.2 | -59.6 | 14.7 |
| SG8-7B | 243.8 | -50 | 300 | -58.6 | 3.1 |
| SG9-1A | 233.4 | -45.6 | 285.6 | -63 | 14.9 |
| SG9-2A | 259.6 | -63.7 | 328.5 | -54.6 | 11.7 |
| SG9-3A | 229.6 | -46.9 | 285.3 | -65.9 | 9.1 |
| SG9-4A | 240.5 | -55.7 | 309.6 | -62.3 | 9.8 |
| SG9-5A | 238 | -50.3 | 297.6 | -62.2 | 9.8 |
| SG9-6A | 237.4 | -41.9 | 282.2 | -58.6 | 7.8 |
| ZL1-1A | 354.4 | 19 | 334.3 | -51.5 | 2.8 |
| ZL1-2A | 0.8 | 15.3 | 335.6 | -58.6 | 0.5 |
| ZL1-3A | 356.2 | 20.5 | 338 | -51.9 | 2.3 |
| ZL1-4A | 359.9 | 18.1 | 338.6 | -56.1 | 0.8 |
| ZL1-5A | 359.7 | 17.7 | 337.8 | -56.3 | 0.7 |

| | | | | | |
|---|---|---|---|---|---|
| ZL1-7A | 356.7 | 13.7 | 328.9 | -56.3 | 1.6 |
| ZL1-8A | 1.8 | 15.2 | 336.6 | -59.4 | 0.7 |
| ZL1-9A | 3.2 | 15.1 | 338.4 | -60.5 | 0.1 |
| ZL2-1A | 197.2 | 6.3 | 105 | 82.1 | 7.6 |
| ZL2-1B | 193.4 | 9.5 | 93 | 77.6 | 5.9 |
| ZL2-3A | 199 | 8.5 | 84.7 | 83.1 | 6.7 |
| ZL2-3B | 198.4 | 4.8 | 116.2 | 83.4 | 2.3 |
| ZL2-5A | 190.7 | -0.1 | 134.2 | 74.9 | 11.7 |
| ZL2-5B | 188 | 29.4 | 56.4 | 60.8 | 10.7 |
| ZL2-5C | 188.2 | 22.8 | 65.7 | 65.9 | 3.3 |
| ZL3-1A | 7.5 | 14.8 | 344 | -63.7 | 0.9 |
| ZL3-2A | 7.7 | 14.6 | 344.1 | -64 | 1.4 |
| ZL3-3A | 6.6 | 14.1 | 341.5 | -63.5 | 0.8 |
| ZL3-4A | 7.6 | 14.2 | 343.1 | -64.2 | 0.8 |
| ZL3-5A | 7.2 | 13.7 | 341.9 | -64.3 | 1.2 |
| ZL3-6A | 7 | 13.2 | 340.7 | -64.5 | 1.1 |
| ZL3-7A | 12.8 | 14.6 | 353.6 | -67 | 0.3 |
| ZL3-10A | 6.3 | 17 | 345.5 | -61.3 | 0.9 |
| ZL3-11A | 0.3 | 19 | 340.2 | -55.8 | 1.4 |
| ZL4-1A | 124.1 | 11.2 | 104.4 | 9.9 | 2.6 |
| ZL4-2A | 122.1 | 14.8 | 100.7 | 8.2 | 3.2 |
| ZL4-3A | 119.6 | 11.7 | 103.7 | 5.5 | 4.9 |
| ZL4-4A | 121.4 | 8.7 | 106.8 | 7.1 | 3.1 |
| ZL4-5A | 114.7 | 18 | 97.1 | 1.2 | 4.4 |
| ZL4-6A | 138.9 | 11.1 | 105.1 | 24.4 | 4.2 |
| ZL4-7A | 291.3 | -5.9 | 288.8 | 3.1 | 2.5 |
| ZL4-8A | 121.1 | 11.7 | 103.8 | 7 | 2.8 |
| ZL5-3A | 186.2 | -10 | 153.8 | 66 | 6.4 |
| ZL5-6A | 185.8 | 1.7 | 124.2 | 70.5 | 8.9 |
| ZL5-7A | 174.1 | 2.5 | 118.4 | 59.1 | 12.8 |
| ZL5-9A | 191.9 | -2.4 | 144.3 | 75 | 9.2 |
| ZL5-10A | 182.3 | 5.9 | 111.7 | 67.4 | 12.6 |
| ZL5-10B | 197.1 | 2.2 | 134.6 | 81.6 | 5.7 |
| ZL7-1A | 192.2 | -5.1 | 133.2 | 72.8 | 1.6 |
| ZL7-2A | 182.8 | -7.8 | 134.4 | 63 | 7.1 |
| ZL7-3A | 189.6 | -5.5 | 132.6 | 70.1 | 4.4 |
| ZL7-4A | 183.1 | -10.6 | 140.4 | 62.6 | 7.1 |
| ZL7-5A | 184.2 | -7.6 | 134.5 | 64.4 | 4.2 |
| ZL7-6A | 185.8 | -7.5 | 135.4 | 65.9 | 4.2 |
| ZL7-7A | 181 | -6.7 | 131.4 | 61.5 | 2.9 |
| ZL7-8A | 189.3 | -5.4 | 132 | 69.8 | 2.4 |
| ZL8-1A | 352.9 | 45.7 | 358.8 | -35.2 | 3.9 |
| ZL8-3A | 331.1 | 49.4 | 352.8 | -21.1 | 4.1 |
| ZL8-4A | 341 | 47.6 | 354.5 | -27.7 | 6.2 |

| | | | | | |
|---|---|---|---|---|---|
| ZL8-5A | 346.3 | 46.7 | 356.1 | -31.1 | 2.6 |
| ZL8-6A | 353.3 | 42.5 | 356.1 | -37.6 | 1.6 |
| ZL8-7A | 356 | 39.9 | 355.4 | -40.9 | 3.4 |
| ZL9-1A | 193.8 | -8 | 144.4 | 73.3 | 3.9 |
| ZL9-2A | 193.7 | -11.6 | 154.4 | 71.5 | 8.3 |
| ZL9-3A | 190.8 | -12.2 | 151.5 | 68.8 | 4.8 |
| ZL9-4A | 192.3 | -11.5 | 151.9 | 70.4 | 2.6 |
| ZL9-5A | 195.9 | -7.4 | 145.3 | 75.5 | 5.1 |
| ZL9-6A | 193.3 | -10.2 | 150 | 71.9 | 3 |
| ZL9-7A | 193 | -10.7 | 150.9 | 71.3 | 2 |
| ZL9-8A | 192 | -10 | 147.5 | 70.8 | 2.1 |
| ZL9-9A | 189.6 | -15.4 | 157 | 66 | 3.5 |
| ZL9-10A | 190.2 | -9.2 | 143.3 | 69.5 | 2.2 |
| ZL10-1A | 192.2 | -14.7 | 159.3 | 68.5 | 3.2 |
| ZL10-2A | 189.7 | -2.6 | 123.9 | 70.7 | 4.5 |
| ZL10-3A | 190.7 | -16.5 | 160.6 | 66.2 | 3.6 |
| ZL10-4A | 194.4 | -15 | 163.8 | 69.9 | 4.6 |
| ZL10-5A | 190.6 | -12.8 | 152.6 | 68.3 | 1.4 |
| ZL10-6A | 191.5 | -13.1 | 154.8 | 68.9 | 2.1 |
| ZL10-7A | 192.1 | -12.5 | 154.3 | 69.7 | 5.9 |
| ZL10-8A | 183.2 | -5.8 | 130.2 | 63.8 | 6.5 |
| ZL11-1A | 182.6 | 27.8 | 65 | 64.5 | 14.5 |
| ZL11-1B | 186.3 | 29.8 | 56.4 | 65.4 | 14.3 |
| ZL11-2A | 168.8 | 13.4 | 102.8 | 57.4 | 13.9 |
| ZL11-4A | 179.7 | 23.1 | 77.6 | 65 | 14.4 |
| ZL11-5A | 181.5 | 23.8 | 74.2 | 66.1 | 14.5 |
| ZL11-6A | 182.6 | 16.5 | 90.9 | 70.1 | 9.1 |
| ZL11-7A | 186.1 | 23.2 | 68.9 | 69.9 | 9.4 |
| ZL11-9A | 196.1 | 25.5 | 41 | 73.5 | 6.1 |
| ZL12-1A | 178.8 | 6.9 | 117.9 | 66.9 | 6.2 |
| ZL12-2A | 169.1 | 8.7 | 111.4 | 57.6 | 5.7 |
| ZL12-3A | 183.5 | 17.5 | 87.3 | 70.5 | 4.2 |
| ZL12-4A | 173 | 2.5 | 124.7 | 60.2 | 5.1 |
| ZL12-5A | 176.3 | 12.6 | 103.6 | 64.7 | 6.6 |
| ZL12-6A | 176.7 | 10.4 | 108.9 | 65.1 | 4.6 |
| ZL12-7A | 174.7 | 9.4 | 110.8 | 63.1 | 6.8 |
| ZL12-9A | 177 | 9.3 | 111.6 | 65.3 | 5.2 |
| ZL13-1A | 24.9 | 17.4 | 84.5 | 75.2 | 9.9 |
| ZL13-2A | 33.5 | 17.3 | 92.2 | 67.4 | 7.1 |
| ZL13-3A | 28.2 | 22.1 | 103.8 | 73.2 | 14.2 |
| ZL13-4A | 27 | 27.4 | 122.7 | 73.7 | 3.5 |
| ZL13-6A | 36.1 | 31.9 | 128.8 | 64.8 | 7.7 |
| ZL13-6B | 52.8 | 25.3 | 113.4 | 50.8 | 8.7 |
| ZL13-7A | 46.3 | 21.8 | 106.6 | 56.4 | 10.9 |

| | | | | | |
|---|---|---|---|---|---|
| ZL14-2A | 29.7 | 31.3 | 132 | 70.1 | 4.4 |
| ZL14-3A | 29.6 | 33.3 | 137.3 | 69.3 | 2.9 |
| ZL14-4A | 38.5 | 22.6 | 106.8 | 63.7 | 6.5 |
| ZL14-5A | 32.6 | 24.8 | 112.1 | 69.1 | 6.4 |
| ZL14-6A | 59.5 | 29.5 | 120.2 | 45.2 | 9.4 |
| ZL14-7A | 21 | 31.5 | 146.5 | 76.4 | 2.6 |
| ZL14-8A | 30.6 | 26.3 | 117.1 | 70.8 | 9.2 |
| ZL15-1A | 36 | 22.7 | 106.7 | 66 | 5.1 |
| ZL15-2A | 27.9 | 28.9 | 126.7 | 72.4 | 2.2 |
| ZL15-2B | 36.6 | 23 | 107.5 | 65.5 | 2.9 |
| ZL15-3A | 34.6 | 37 | 140.7 | 64 | 1.3 |
| ZL15-4A | 23.5 | 32 | 142.8 | 74.4 | 7.9 |
| ZL15-7A | 36.7 | 29.4 | 122.6 | 64.9 | 2.8 |
| ZL15-9A | 35.9 | 30.7 | 125.9 | 65.3 | 5.6 |
| ZL19-1A | 47.2 | 20.1 | 103.8 | 55.3 | 5.3 |
| ZL19-2A | 53.8 | 17.9 | 102.4 | 48.8 | 4.1 |
| ZL19-3A | 50.9 | 19.6 | 104.1 | 51.8 | 3.4 |
| ZL19-4A | 50.7 | 18.9 | 103 | 51.8 | 5 |
| ZL19-5A | 54.3 | 18.1 | 102.9 | 48.4 | 3.1 |
| ZL19-6A | 52.5 | 18.1 | 102.3 | 50 | 5.2 |
| ZL19-8A | 51 | 22.1 | 108.1 | 52.2 | 8.2 |
| ZL19-9A | 50.9 | 19.7 | 104.2 | 51.8 | 8.1 |
| ZL19-10A | 49 | 26.1 | 114.5 | 54.3 | 9.3 |
| ZL20-1A | 48.2 | 18.1 | 100.9 | 54 | 1.3 |
| ZL20-1B | 43.1 | 11.1 | 86.3 | 56.6 | 5 |
| ZL20-2A | 42.9 | 13.1 | 89.7 | 57.4 | 2.8 |
| ZL20-3A | 41 | 13.9 | 89.9 | 59.4 | 5.8 |
| ZL20-4A | 41.6 | 11.7 | 86.4 | 58.1 | 2.7 |
| ZL20-5A | 45 | 15.9 | 95.7 | 56.4 | 14.6 |
| ZL20-6A | 43.6 | 12.8 | 89.6 | 56.8 | 2.1 |
| ZL20-7A | 42.8 | 14.4 | 92 | 58 | 4.4 |
| ZL20-8A | 44 | 12.2 | 88.8 | 56.2 | 4 |
| ZL20-9A | 43.7 | 12.4 | 89 | 56.5 | 2.8 |
| ZL21-1A | 29.9 | 11.4 | 74.1 | 68.2 | 3.7 |
| ZL21-1B | 30.4 | 17.9 | 91.5 | 70.4 | 4 |
| ZL21-1C | 29.4 | 16.1 | 85.5 | 70.7 | 4.2 |
| ZL21-2A | 29 | 15.8 | 84.4 | 71 | 3.7 |
| ZL21-3A | 32.5 | 13.5 | 82.2 | 67 | 2.6 |
| ZL21-3B | 34 | 16.6 | 90.8 | 66.8 | 5.9 |
| ZL21-4A | 27.3 | 11.4 | 70.2 | 70.3 | 3.8 |
| ZL21-4B | 33.7 | 20.6 | 100.8 | 67.9 | 4.8 |
| ZL22-1A | 43.3 | 7.7 | 81.1 | 54.9 | 4.5 |
| ZL22-1B | 46.2 | 12.8 | 91.2 | 54.4 | 6.8 |
| ZL22-2A | 39.3 | 18.9 | 98.9 | 62.5 | 10.7 |

| | | | | | |
|---|---|---|---|---|---|
| ZL22-3A | 46.8 | 11.3 | 89.1 | 53.3 | 9.5 |
| ZL22-4A | 47.3 | 12.9 | 92 | 53.4 | 5.1 |
| ZL22-5A | 39 | 12.5 | 85.8 | 60.8 | 4.3 |
| ZL22-6A | 38.2 | 15.6 | 91.4 | 62.6 | 3 |
| ZL22-7A | 45.9 | 9.1 | 85.2 | 53.2 | 5.1 |
| ZL23-1A | 47.8 | 14.9 | 95.4 | 53.5 | 5.1 |
| ZL23-2A | 48.3 | 13.5 | 93.4 | 52.6 | 8.9 |
| ZL23-3A | 46.4 | 18.4 | 100.6 | 55.7 | 11.7 |
| ZL23-5A | 46.6 | 15 | 95 | 54.7 | 2.8 |
| ZL23-6A | 42.6 | 13.3 | 89.9 | 57.8 | 4.9 |
| ZL23-7A | 47.9 | 16.1 | 97.4 | 53.7 | 7.2 |
| ZL23-8A | 43.2 | 22.5 | 107.4 | 59.3 | 9 |
| ZL23-9A | 44 | 19.9 | 102.6 | 58.2 | 12 |
| ZL23-10A | 42.8 | 22.3 | 106.9 | 59.7 | 9.3 |

*Notes:* Dg and Ig, declination and inclination in geographic coordinates; Ds and Is, declination and inclination in stratigraphic coordinates; MAD, mean angular deviation of the ChRM fit; the same sampling core named with A and B underwent stepwise thermal or alternating field demagnetization.
